# Bacterial microbiome associated with cigarette beetle *Lasioderma serricorne* (F.) and its microbial plasticity in relation to diet sources

**DOI:** 10.1371/journal.pone.0289215

**Published:** 2024-01-19

**Authors:** Thanga Suja Srinivasan, Krishnamanikumar Premachandran, Paul X. Clinton

**Affiliations:** Centre for Climate Change Studies, International Research Centre, Sathyabama Institute of Science and Technology, Chennai, Tamil Nadu, India; Central Rice Research Institute: ICAR - National Rice Research Institute, INDIA

## Abstract

Insect-microbial symbiosis contributes positively to the physiology of the insect and diet is considered as one important factor determining microbial symbiosis. In this study, we have characterized the microbiota of cigarette beetle, *Lasioderma serricorne* (Fabricius) on different diets and phases. The beetles were reared on different diet sources (exposed phase) for six generations and were reverted to their natal source (reverted phase) and further maintained for six more generations. The bacterial diversity and richness were higher in the exposed phase and once reverted to the natal source, the microbial abundance has re-assembled according to the natal diet source. There was re-assemblage of microbial composition in accordance to the diet and the bacterial cells are able to establish and proliferate on reverting to their natal source. The bacterial composition of the beetle was mainly dynamic and not transient where the bacterial cells were maintained at low abundance and were re-established according to the diet source. Overall, we found that the microbiota of cigarette beetle to be dynamic and bacterial composition to re-assemble in a diet-specific manner. The study provides insights on diet associated microbial plasticity of cigarette beetle and a further comprehensive understanding on mechanisms involved in microbial plasticity will help develop novel pest management strategies for this invasive insect pest.

## Introduction

Insect species are one of the most successful groups in animal kingdom and have colonized a variety of terrestrial habitats ranging from hot sand dunes to snow-capped mountains. This is mainly due to their ability to withstand adverse conditions and efficiently utilize the resources. Insects have evolved many different strategies including their association with microbial symbionts to extend their ability and adaptability to cope with diverse adverse conditions. Insect microbiota plays an important role in insect biology and is pervasive in almost all body parts and constitutes 1–10% of insect biomass [1]. They have a central role in insect adaptation by facilitating nutrition and immune benefits ultimately resulting in insect fitness [1–3]. The insects provide suitable environment for their microbial partners and transmit them for the offspring’s through horizontal or vertical transmission [4, 5]. On the other hand, microbes aid in host metabolism by breaking down complex polysaccharides [6, 7], provision of essential nutrients, sterols and vitamins from nutrient deficient resources [1], degradation or detoxification of toxic allelochemicals and insecticides [1]. The grain pest beetle *Oryzaephilus surinamensis* with its intracellular bacteroidetes symbionts (*Candidatus* Shikimatogenerans silvanidophilus) supplement the beetle with tyrosine precursors and this confers structural protection of the beetle against desiccation and predation [3]. Elimination of these symbiotic partners by heat treatment or antibiotics has a significant negative effect on the life history traits of the insect [8, 9]. Although not strictly essential for mere survival of insect, microbe-insect interaction influences many aspects of development, physiology, reproduction and behaviour to ecology of the insect [5]. Aposymbiotic insects exhibit decrease in nymphal development and adult emergence duration in *Brachynema germari*, *Acrosternum heegeri*, and *Acrosternum arabicum* [10], and reproductive potential in *Graphosoma lineatum* [9].

Host diet is one among an important factor shaping the gut microbiota of insects [1, 11, 12] i.e., termites [13]; *Drosophila* [14]; cockroaches [15]; moths [16–18] flies [19] etc. A change in diet can alter the microbiota and thereby influence insect fitness instantly [17]. The metagenomics studies on termites revealed the community structure and functional potential of termites to be strongly driven by the digestive strategy of the host [13]. The gut microbiota of cockroach (*Blattella germanica)* was highly dynamic, and was reassembled by host specific diet in a short time span [15]. This microbial plasticity can be quite useful for insects to exploit diverse food sources which serves as a base for evolution of host associated biotypes/populations [1, 5, 18]. For example, the bacterial symbiont community of brown planthopper (*Nilaparvata lugens*) varied among biotypes reared on different host plants [20].

Cigarette beetle, *Lasioderma serricorne* (Fabricius), (Coleoptera: Anobiidae), is a worldwide pest of stored products. It is a polyphagous insect and can survive and adapt to food sources ranging from edible (whole wheat flour, soybean flour, spices and condiments, cotton seeds, dried yeast, biscuits, chocolates) to non-edible produces like herbarium specimens, leather and toxin containing compounds like cured tobacco leaves with nicotine and insecticide containing pyrethrum etc. [21, 22]. The beetle has four life stages (egg, larva, pupa, adult) and the larval stage is the most active feeding stage inflicting damage to stored products The adult beetles are devoid of feeding but makes holes trying to escape or enter packed products, inflicting lesser damage than larva. The dynamics of cigarette beetle population is highly influenced by many environmental factors like food source, light, temperature, and relative humidity [21]. Cigarette beetles transmit their yeast like endosymbiont through vertical transmission by egg smearing where newly hatched larva feed on the egg shells to receive symbionts. *Symbiotaphrina kochii* is a primary yeast-like symbiont of cigarette beetle involved in B-vitamin biosynthesis, fatty acid metabolism, and detoxification of toxic phytochemicals [21–23]. However, the bacterial microbiota of cigarette beetle and changes in bacterial community structure on shift in food source remains unexplored.

In the study, we propose that cigarette beetles exhibit microbial plasticity in accordance with their diet source. Any change of diet exhibits a re-assemblage of structure or abundance of the beetle microbiome and once the beetles are again reverted to their original natal source the microbial composition and structure is reverted back. To test our hypothesis, we have cigarette beetle from three different phases i.e., natal, exposed and reverted colonies. Initially, we examined the overall bacterial community structure associated with cigarette beetle. Then, we looked for change in abundance of bacterial community structure among the diet samples in different phases to detect significant food source effects. To further test the hypothesis, we examined the reverted and natal phase for any significant shift in bacterial taxa. This study on bacterial symbiosis of cigarette beetle can further improve our understanding on the symbiont associated evolutionary success of the beetle on different food sources.

## Materials and methods

### Insect rearing

Cultures were initiated with twenty to thirty *L*. *serricorne* (F.) species collected from infested wheat flour from a storage godown, at Chengalpattu district, Tamil Nadu (12.693933° N and 79.975662° E). The beetles were transferred to the laboratory the same day and maintained in 500 ml beaker containing 50 grams of wheat flour as food source at 28˚C with 12 hrs of daylight and 12 hrs of darkness. The glass beakers were closed with mylar cloths for better air circulation. After one population cycle, (approximately 45 days in natal wheat) the beetles were transferred to a new 500 ml beaker containing 50 grams of wheat flour as the food source and were further maintained on wheat source for six generations and were used in the study. The wheat reared beetles will be hereafter in the study called as natal colonies or natal phase. The food sources employed in the study were kept under UV light for 15 to 20mins before beetle infestation to avoid possible contamination from food sources.

### Acclimatization to different food sources

**Fig 1:**
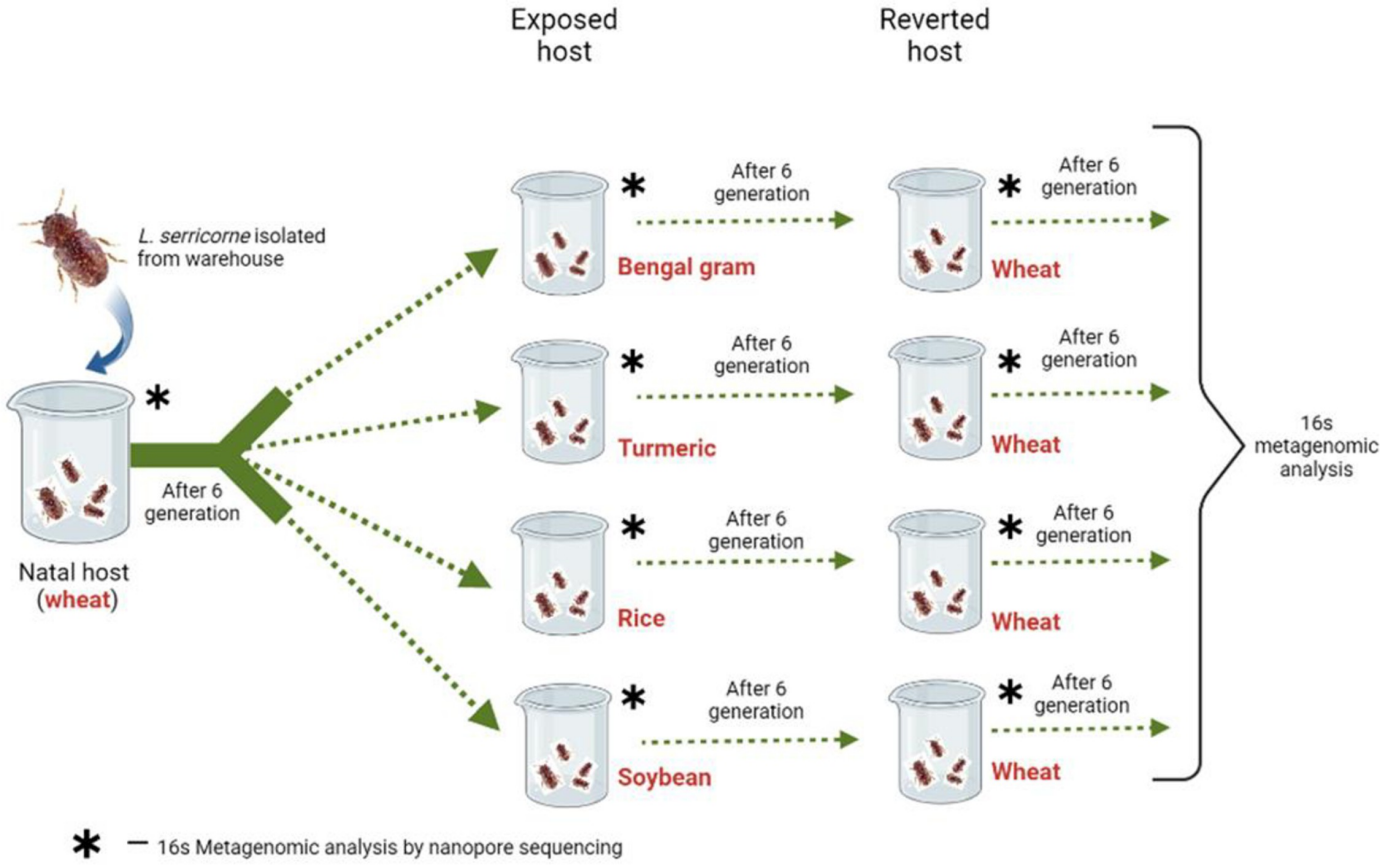
Details of the experiments with cigarette beetle and microbiome sampling. Cigarette beetle were initially cultured on wheat as natal (N) source (called as natal phase) for 6 generations (G) and sampled for microbiome analysis at N- 6G. Then natal phase colonies were cultured for another 6G on each of exposed (E) diets called as exposed phase colonies and beetles sampled for microbiome at E-6G. The colonies were continued for a further 6G on wheat diet called as reverted (R) phase with microbiome sampling at R-6G. Six biological replicates were maintained for each treatment and three technical replicates for 16s DNA sequencing.

Ten beetles of the same stage from the original natal colony were transferred to four different food sources each (rice flour, turmeric powder, Bengal gram (whole desi brown seed coat type), soybean flour) hereby called as exposed colonies/phase. They were maintained on the exposed food sources in 500 ml glass beaker with 50 grams of food source each under the above said conditions. Six replicates were maintained for each colony in exposed phase. Once the individual exposed colonies had completed one population cycle, they were further shifted to the same exposed source for the subsequent six generations without selection. The generation time varied in exposed food sources and is mentioned in S1 Table. After culturing six generations in exposed food sources, adult beetles were collected randomly from individual colonies and stored at -80ºc until further use. The remaining beetles at different stages were again reverted/introduced to natal source wheat hereafter called as reverted colonies/phase with 50 grams of wheat in 500 ml beaker at the above said conditions. The generation time of reverted colonies varied for initial 2–3 generations, after which the generation time was approximately 45 days like natal source. After six generations of life cycle as reverted colonies without selection, the adult beetles were collected randomly and stored at -80ºc until further use. Six replicates were maintained for each colony in reverted phase. A detailed diagrammatic representation of the experiment is given in Fig 1.

### Nanopore sequencing and data analysis

Three uniformly sized female adults (approximately 0.5 mg each) were randomly selected from each replicate and subjected for DNA extraction using Qiagen DNeasy Blood & Tissue kit. The beetles were surface sterilized with 70 per cent ethanol for 60 secs followed by rinse in sterile distilled water three times before DNA extraction. The above procedure was carried under a sterile hood. After DNA extraction, the replicates were pooled together to create a representative DNA sample. And from the representative sample three technical replicates were drawn and subjected for PCR amplification by 16s rRNA primers (Forward 16S primer: 5`-AGAGTTTGATCMTGGCTCAG-3`; Reverse 16S primer: 5`-CGGTTACCTTGTTACGACTT- 3`). The amplified product length of 1500 bp was checked and purified further by using 1.6X Ampure XP beads (Beckmann Coulter, USA). Library preparation was carried out and nanopore sequencing was performed on GridION X5 (Oxford Nanopore Technologies, Oxford, UK) using SpotON flow cell R9.4 (FLO-MIN106) in a 48 hrs sequencing protocol. Nanopore raw reads (‘fast5’ format) were base-called (‘fastq5’ format) and de-multiplexed using Guppy v2.3.4. The Nanopore raw reads were processed using Porechop [24]. After trimming, on average, 134006 reads per sample (maximum = 194193; minimum = 89575) passed and were retained for bacterial identification. Centrifuge tool was used to align the processed reads against Centrifuge-P bacterial database for bacterial classification which is a very rapid and memory- efficient system for microbial samples with better sensitivity and accuracy [25]. The system uses a novel indexing scheme based on the Burrows Wheeler transform (BWT) and the Ferragina-Manzini (FM) index, optimized specifically for the metagenomic classification problem [25]. The data from Centrifuge tool in kreport format was used for Pavian web application to explore the taxonomic content for all samples and the output was in excel format. Pavian, web application was mainly used to estimate and interactively explore the taxonomic content [25]. Sequences were submitted in the NCBI SRA database linked to the NCBI Bio-Project accession number PRJNA792026.

### Data analyses

The classification of microbiome along with the abundance values obtained as output file in excel format was used for further analysis. Clear asymptotes were observed in rarefaction curve analyses indicating a near-complete sampling of the bacterial communities (S1 Fig). Alpha diversity indices were calculated by microbiome analyst online (https://www.microbiomeanalyst.ca/), which showed the complexity of microbiome across diets and phases. Kruskal–Wallis one-way analysis of variance was performed to compare alpha diversity estimates and p < 0.05 was considered statistically significant. Beta diversities analysis was performed and principal coordinate analyses (PCoA) based on abundance were determined, in order to investigate structural variation in microbial communities of phases and diets using weighted UniFrac distance metrics (Lozupone and Knight, 2005). Nonmetric multidimensional scaling (MDS) was used to visualize microbiota composition among phases. Heat map of 35 predominant families in the microbial communities was constructed in Orange web tool https://orangedatamining.com/widget-catalog/visualize/heatmap/ with diet samples across column and relative abundance of families across rows. Venn diagram was developed to observe the share of genus taxa among phases in individual diet sources using online web tool https://bioinformatics.psb.ugent.be/webtools/Venn/.

Permutational analysis of variance (PERMANOVA: Anderson, 2001) was used to analyse differences in microbiota composition across diets and phases. The fixed factors included in the permanova analysis were “diet” and “phases”. Permanova pair wise test results were performed among factor levels, “diet” and “phases”. The PERMDISP was done to examine homogeneity of dispersions and was made sure the permanova results were not affected by data dispersion. Similarity, percentage analysis (SIMPER) was used to identify genus taxa that has contributed most to the dissimilarities between phases and diets.

The microbial composition of cigarette beetle across diets and phases were analysed using univariant and multivarant GLM. The proportional and relative abundance of taxa genus was examined for change in microbiome composition across diet sources and phases using multivariant GLM. The genus *Wolbachia*, was excluded from proportional abundance as it can mask the effect of other genera and the remaining predominant taxa that has contributed ≥ 0.12% to the data were included. The most abundant *Wolbachia* genera was subjected to univariant GLM across diets and phases. To restrict our focus with genus that has contributed to the change in abundance across phases, only taxa genus that were significantly different across phases were chosen. An univariant GLM analysis was performed on selected taxa genus among wheat and reverted diet samples alone.

Univariant and multivariant GLM were statistically performed using SPSS v.22. PERMANOVA, PERMDISP, nMDS, PCoA and SIMPER analysis were carried out using PRIMER software (v.7) with PERMANOVA +add on.

## Results

### Distribution of taxa and phylotypes

**Fig 2:**
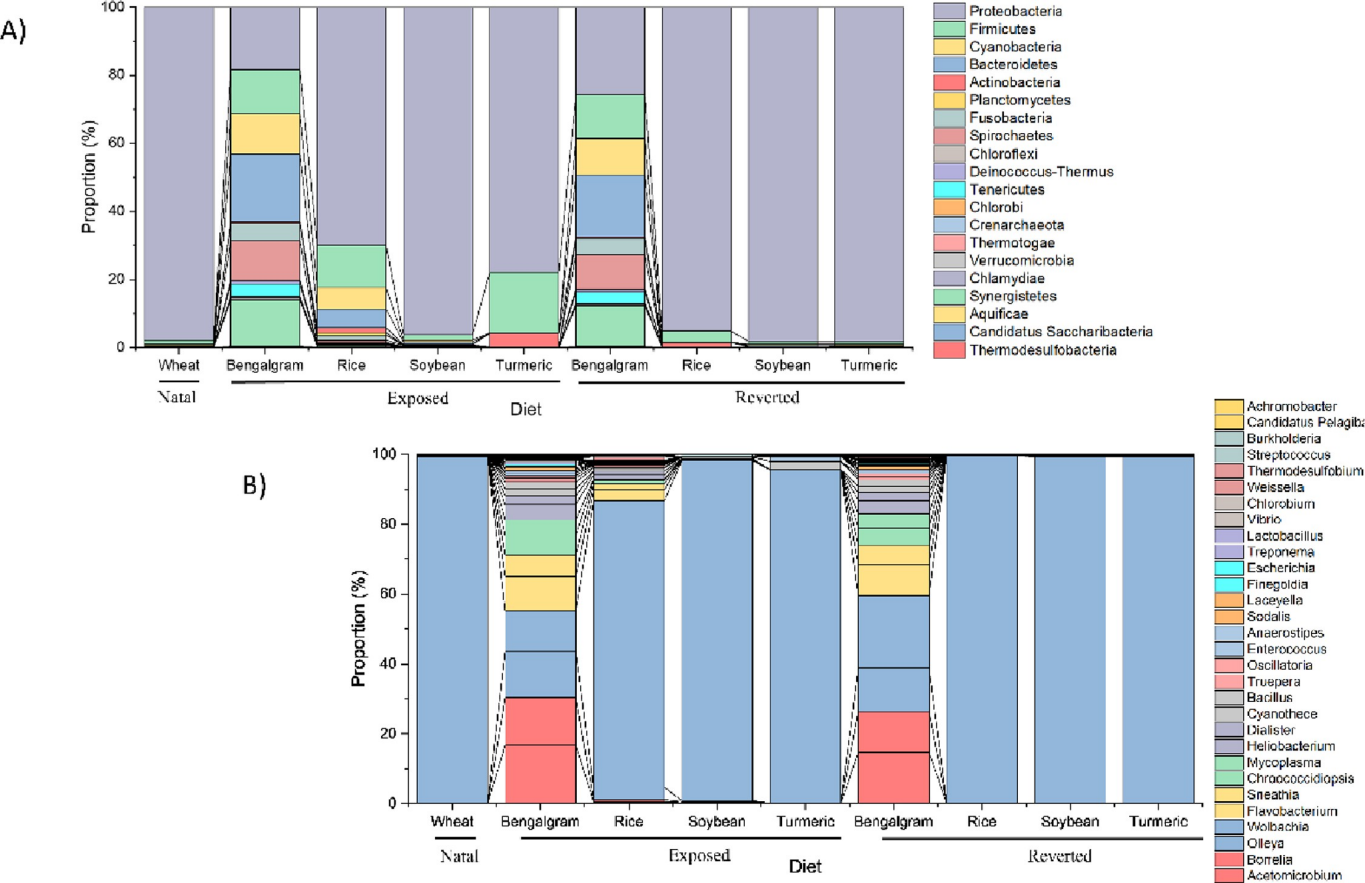
Bacterial structure of cigarette beetle microbiome at phylum and genus level. Bar-plots show the abundance and distribution of the A) 20 most abundant phylum and B) 30 most abundant genera across diets.

The microbial community composition of cigarette beetle was obtained by nanopore sequencing across diet samples from three phases. A total of ∼100000 to ∼200000 reads were generated from the samples and the average read length was ∼1500bp. The average median read length (N50 value) was ∼1650bp which indicates better genome assemblies. Unclassified reads from raw reads were below 4% except rice (exposed) which had ∼7%. The taxa which were observed in less than 9 samples and their total abundance less than 11 were removed from further analysis in order to minimize artefacts. The detected microbiome was classified into 39 phyla, 75 classes, 155 order, 319 families, 826 genera and 1731 species. Proteobacteria (79.2%) was the most predominant phyla followed by Firmicutes (6%), Bacteroidetes (4.1%) and Cyanobacteria (2.9%) whereas the rest of the phyla occupied less than 8%. The phylum Proteobacteria (79.2%) was predominantly represented by classes Alpha Proteobacteria (97.2%), Gamma Proteobacteria (1.8%), Beta Proteobacteria (0.6%) and Delta Proteobacteria (0.2%). In particular, Proteobacteria were predominant in all samples (< 95%) except Bengal gram diet where Proteobacteria has contributed only 18.3% and 25.8% in exposed and reverted phases of Bengal gram (Fig 2 and S2 Table). The proportion of Proteobacteria in exposed phases especially rice and turmeric were lower than their corresponding reverted phases. The distribution of phylum was diverse in Bengal gram exposed and reverted diet where the phylum Bacteroidetes, Proteobacteria, Synergistes, Firmicutes, Cyanobacteria and Spirochaetes have contributed together 87% and 88% in Bengal gram exposed and reverted phases respectively (Fig 2).

### Diversity analysis and microbiome composition

**Fig 3:**
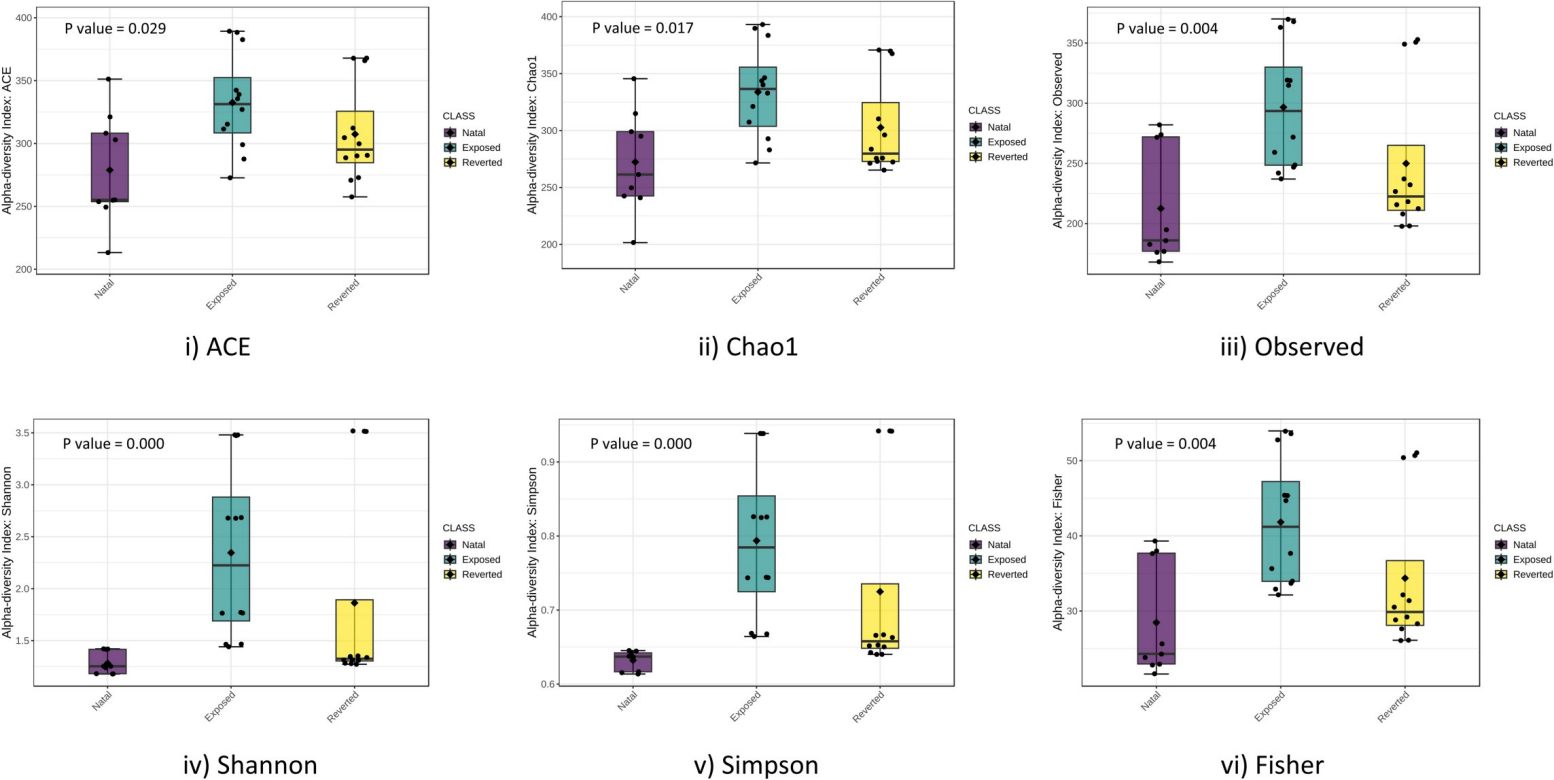
Alpha diversity indices of cigarette beetle microbiome across phases. Statistically significant values * = P ≤ 0.05, ** = P ≤ 0.01, *** = P ≤ 0.005; df = 2.

The microbiome composition of cigarette beetle was examined using alpha diversity indices including observed, Chao evenness, ACE, Shannon, Simpson and Fischer among different diet sources and phases (Fig 3 and S3 Table). Diversity indices indicated that the exposed phase has higher richness and evenness than natal and reverted phases indicating microbial diversity (Fig 3). Similarly, among diet sources Bengal gram and rice (in exposed phase) and Bengal gram to wheat (in reverted phase) had the highest number of taxa evenness and richness compared to other diets indicating the richness of microbiota in these diets (S3 Table).

**Fig 4:**
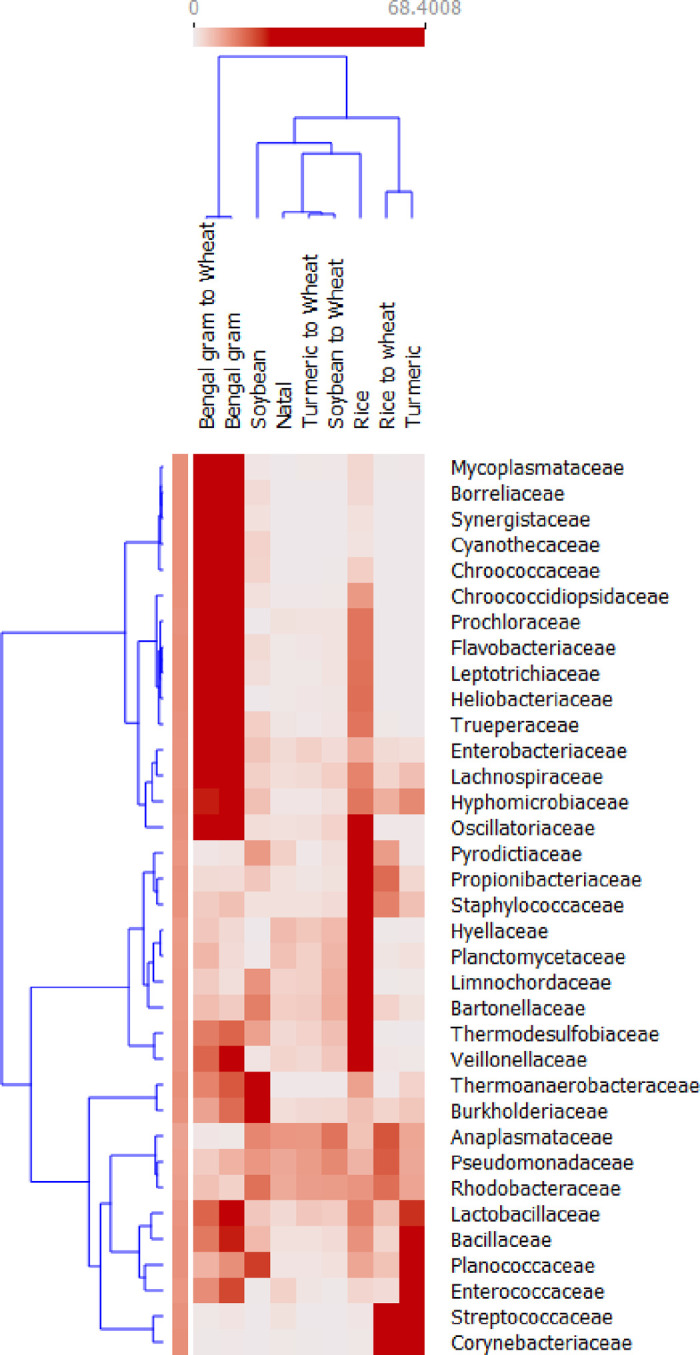
Heat map of the top 35 abundant families showing the relative abundance of the bacteria taxa across diet sources.

**Fig 5:**
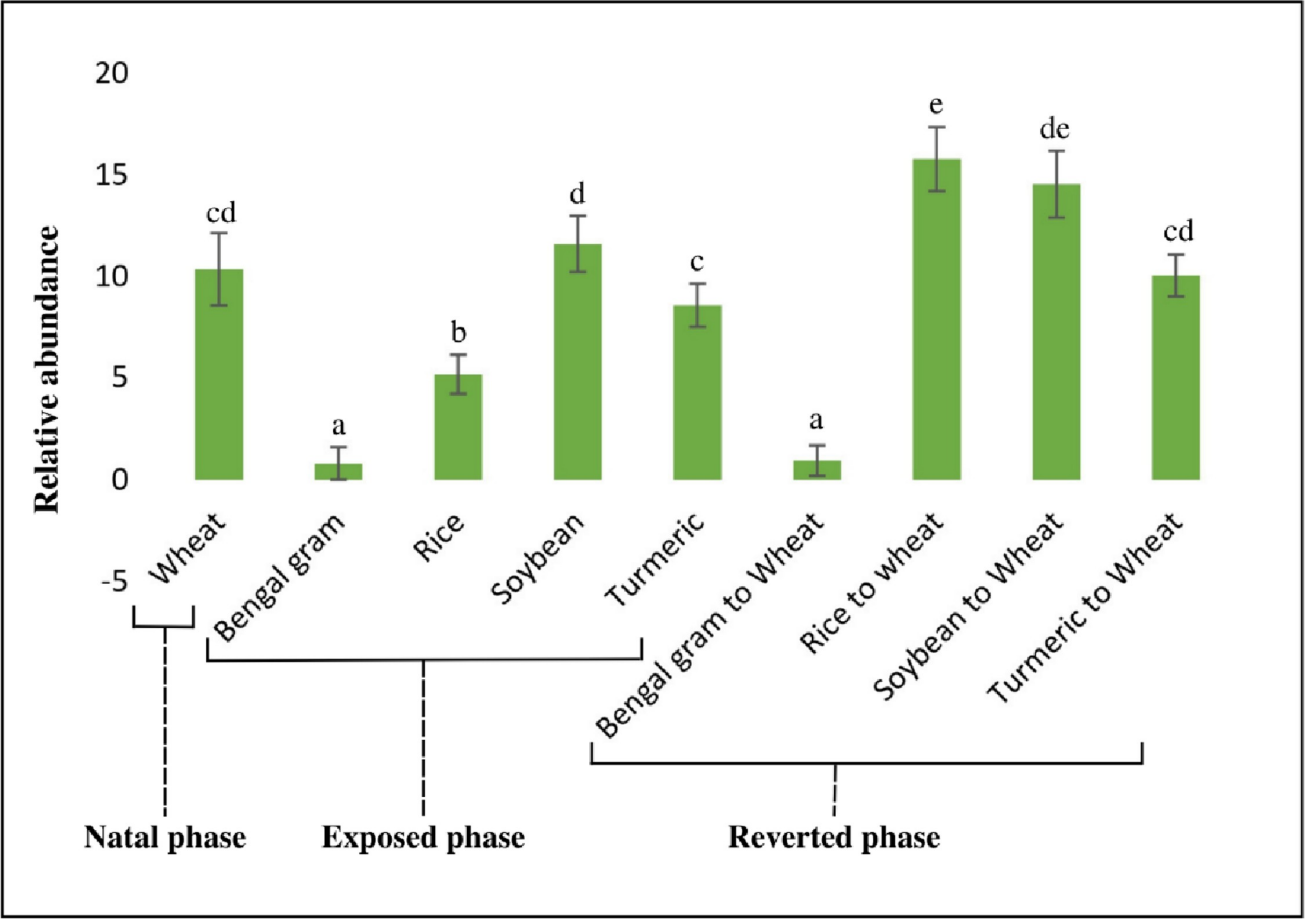
The relative abundance of genus Wolbachia associated with cigarette beetle across diets. Error bars are indicated, lowercase letters indicate homogenous groups (Tukey, p ≤ .05; df = 8).

The microbiota of cigarette beetle was observed at different taxonomic levels. At family level, *Anaplasmataceae* was most prevalent in most of the samples. The most prevalent *Anaplasmataceae* had a significantly lower relative abundance in Bengal gram and Bengal gram reverted diet whereas the relative abundance of *Synergistaceae*, *Cyanothecaceae* and *Borreliaceae* were higher in Bengal gram and Bengal gram reverted to wheat diet than others (Fig 4). The following families showed higher relative abundance in their respective exposed diets but have declined/shifted in abundance on reverting to natal wheat; *Hyellaceae*, *Limnochordaceae*, *Planctomycetaceae*, *Thermodesulfobiaceae*, and *Veillonellaceae* in rice; *Thermoanaerobacteraceae*, *Burkholderiaceae* and *Planococcaceae* in soybean and *Bacillaceae*, *Planococcaceae*, *Enterococcaceae*, *Streptococcaceae* and *Corynebacteriaceae* in turmeric diet (Fig 4). At genus level, *Wolbachia* was most prevalent among all diet sources and contributed 83.8% of the total abundance. On the contrary, very low relative abundances of *Wolbachia* appeared in Bengal gram (0.80 ± 0.03) and Bengal gram reverted to wheat diet (0.94 ± 0.05) (Fig 5) whereas relative abundance of genus *Acetomicrobium*, *Olleya*, *Borrelia*, *Clostridium* and *Flavobacterium* were higher in Bengal gram and Bengal gram reverted diet whereas significantly lower in other diet sources (Fig 2).

**Fig 6:**
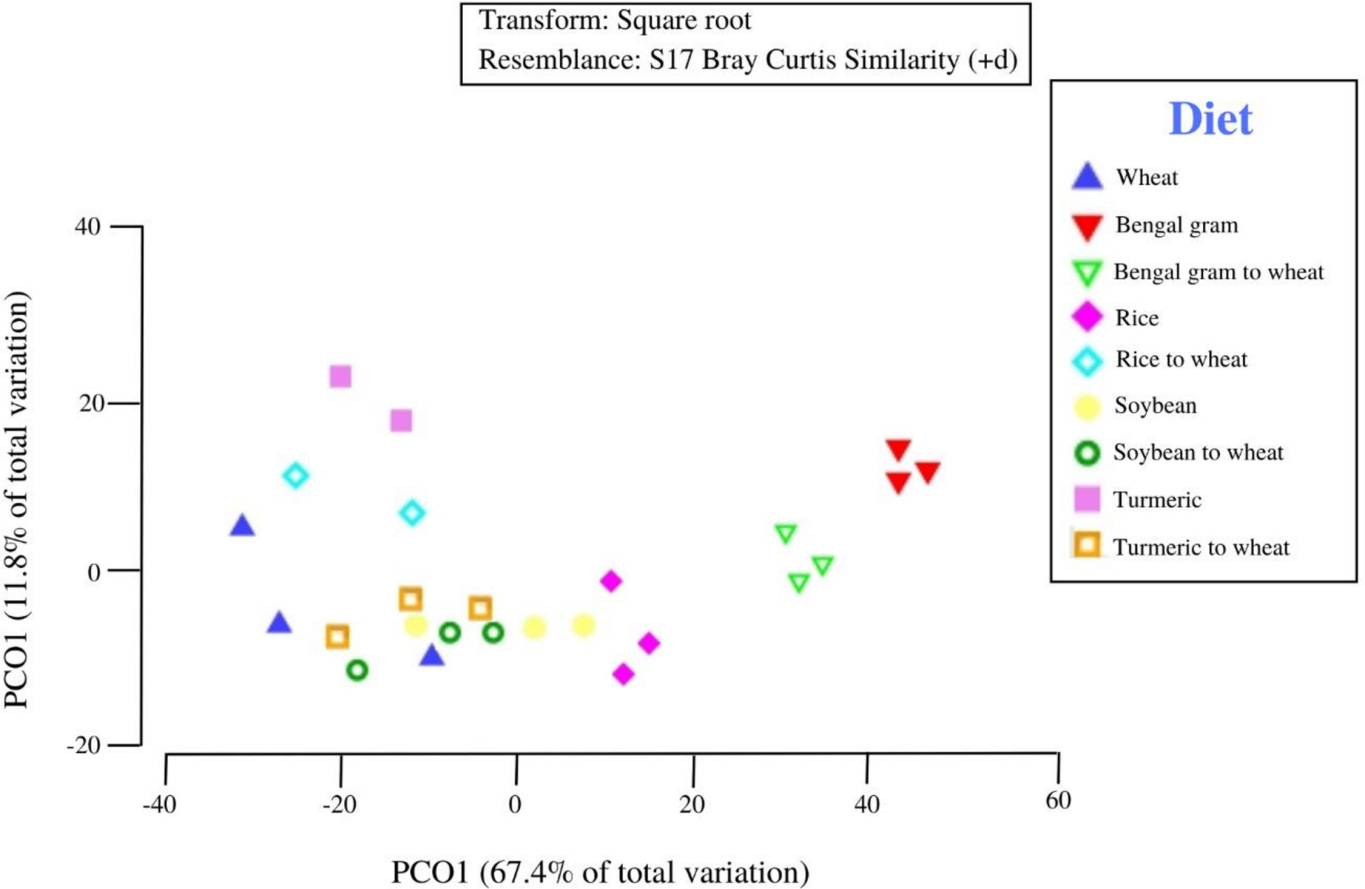
Principal coordinate analysis (PCoA) of community structure of cigarette beetle across diet sources (df = 8) and phases (df = 2). Each symbol represents phase and corresponding color represents diet. Colored triangle represents natal phase; colored inverted triangle, diamond, circle, square represents exposed phase; colorless inverted triangle, diamond, circle and square represents reverted phase.

To evaluate the difference in beta diversity of cigarette beetle from different diet sources and phases. PCoA was used to visualize based on the Bray–Curtis similarities (Fig 6). In the scatter plot, the first two principal coordinates, PCO1 and PCO2, explained 67.4% and 11.8% of the data variation, respectively. Each symbol represents phase and their corresponding color represents diet on the PCoA plot, and most of the diet sources from three phases were distinguishable by PCoA. The natal phase has clustered separately and samples from reverted phase especially soybean and turmeric have clustered relatively close to the natal phase as a separate group which could explain the similarity in microbial composition. Regarding the diet, Bengal gram has clustered separately as a group irrespective of phases whereas the rice and turmeric diet sources have been separated from their respective reverted phase. Interestingly, turmeric and rice in reverted phase has shifted close to the wheat (natal) group (Fig 6) compared to their exposed diets which can explain the re-assemblage and plasticity in cigarette beetle microbiome.

### *Wolbachia* relative abundance

At the genus level, *Wolbachia* genera had abundance 84% in the beetle microbiome and other genera contributed the remaining. To observe the impact of diet and phases on *Wolbachia* abundance a univariate GLM was performed. The relative abundance of genera *Wolbachia* was significantly different among diet sources (*F* = 91.982; *p* = < 0.001) whereas non-significant among phases (*F* = 1.833; *p* = 1.82). Among the diet sources, *Wolbachia* abundance in Bengal gram related diets were significantly lower than other diet sources whereas abundance in wheat related diets were significantly higher stating the *Wolbachia* abundance to be related to diet source (Fig 5).

### Microbiome abundance among diets and phases

At genus level, both phase and diet had a significant impact on the abundance of cigarette beetle microbiome (Pseudo‐*F* = 2.8165, *p* = .018, unique perms = 998; Pseudo‐*F* = 34.255, *p* = .001, unique perms = 999), as shown in the nMDS plot (S2 Fig). PERMANOVA pairwise test results indicated that microbiomes were significantly different between phases, where natal appear to be significantly different from exposed (S2 Fig) (*p(MC)* = 0.034), which is also reflected in the relative size of the pseudo-t statistic (*t* = 1.9327). Similarly exposed and reverted phases exhibited a significant difference (*p(MC)* = 0.041; and *t* = 1.738), whereas natal and reverted phases had no significant difference (*p(MC)* = 0.217, and *t* = 1.234). Similarly, the average pairwise similarity between natal with exposed was lowest (average between group = 61.278) compared to natal with reverted (average between group = 71.222). ANOSIM analysis for phase showed that the average dissimilarities as *R statistics* = 0.159, and the average dissimilarities between natal and exposed phase ranked highest (*R statistics* = 0.326) followed exposed and reverted phase (*R statistics* = 0.139). PERMDISP tests was done for phases which showed non-significant *p*‐values (*F* = 3.10; *p* = 0.21) indicating the PERMANOVA results were not affected by data dispersion.

Similarly, PERMANOVA pairwise tests between levels of the diet factor showed significant differences between wheat and exposed diet samples (all with *p(MC)* values ≤ .05) expect soybean (*p(MC)* = 0.049, and *t* = 2.1026). All the reverted diet samples expect Bengal gram (*p(MC)* = 0.005) and *t* = 3.4089) and rice (*p(MC)* = 0.049, and *t* = 1.8911), exhibited no significant difference with wheat. The average pairwise similarity between wheat with Bengal gram was lowest (average between group distance = 50.87) among all other others. ANOSIM analysis for diet factor showed that the average dissimilarities are higher, *R statistics* = 0.9222, close to its maximum value of 1, and the average dissimilarities between wheat and Bengal gram ranked highest (*R statistics* = 1) followed by rice (*R statistics* = 0.889) and was mainly due to the genera, *Anaplasma* spp. (≥ 2.5 dissimilarity) and *Acetohalobium* spp. (≥ 2.0 dissimilarity) respectively contributing to the dissimilarity (S4 and S5 Tables).

### Re-assemblage of microbiome among diet sources and phases

To visualize the dynamic changes in bacterial communities among diet sources and phases, a dendrogram with heat map was generated using relative abundance of families. Thirty-five most dominant families were used for the analysis. The columns represent the samples and rows represent the bacterial relative abundance assigned to the family level. Congruent with the PCoA analysis, the bacterial communities of Bengal gram and Bengal gram reverted to wheat were similar in composition at the family level and were assigned a separate branch (Fig 4). Rice exhibited a distinct branch whereas the remaining formed a separate branch. The reverted diet sources expect Bengal gram exhibited a decline in abundance of specific taxa`s that were mainly related to their corresponding exposed diet source, ie., rice exhibited shift in abundance related to the families, *Thermodesulfobiaceae*, *Trueperaceae*, *Veillonellaceae*, *Planctomycetaceae*, *Oscillatoriaceae*, *Limnochordaceae*, *Leptotrichiaceae*, *Heliobacteriaceae*, and *Hyellaceae*; in case of turmeric, *Bacillaceae*, *Enterococcaceae*, *Hyphomicrobiaceae*, *Lactobacillaceae*, *Planococcaceae*, *Streptococcaceae* whereas with soybean *Thermoanaerobacteraceae*, *Planococcaceae* were observed when reverted to natal wheat (Fig 4).

**Fig 7:**
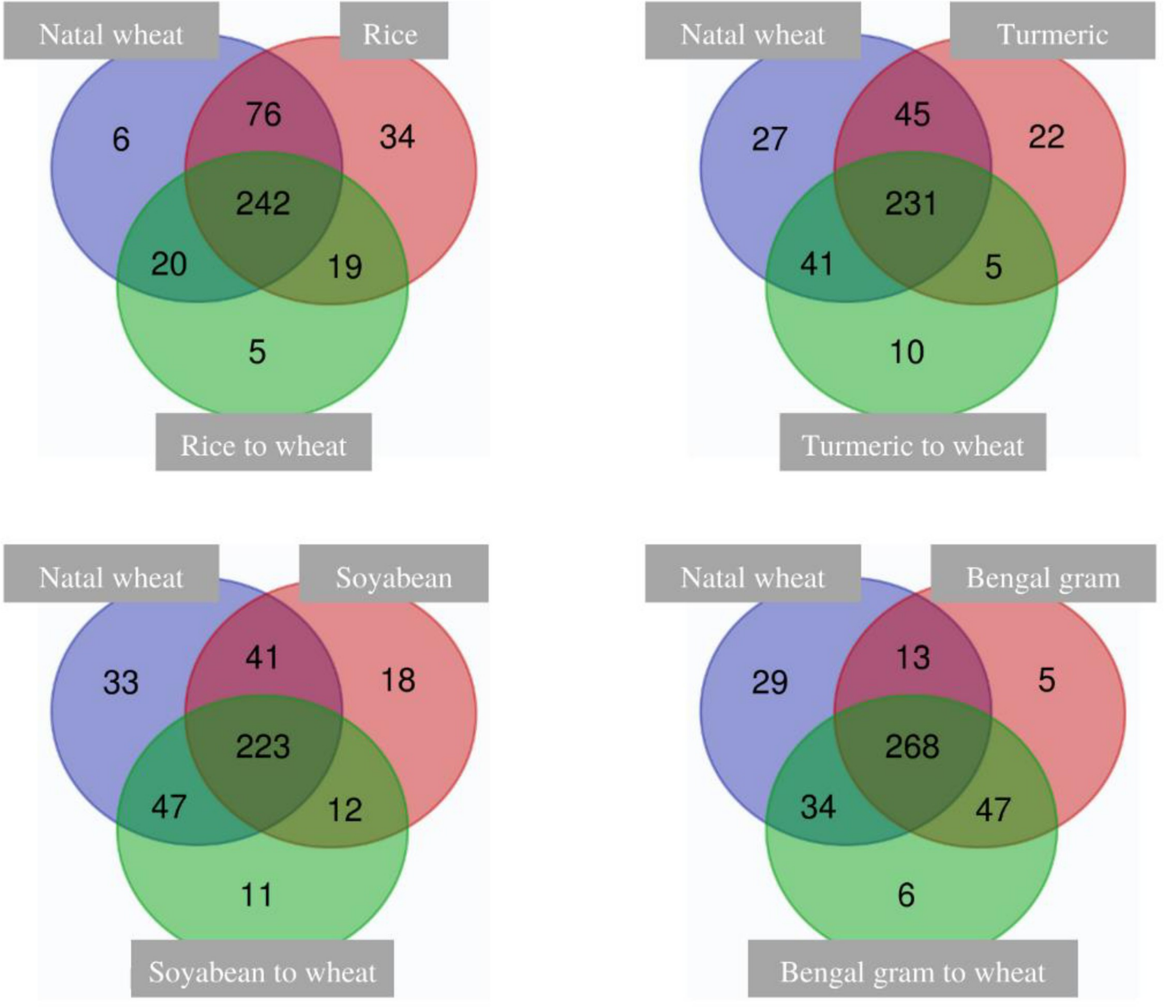
Venn diagram representing the shared genus of cigarette beetle microbiome across diet source.

A total of 115 genera that were present among all diet sources were considered as core taxa and were predominately belonging to Proteobacteria phylum (S6 Table). A venn diagram explaining the shared number of genus indicates natal wheat to Bengal gram has the least number of shared genus (13) along with a higher core share (268) among phases. Similarly, the unique genus counts in Bengal gram is less (5) indicating most of the genus has been transferred to the reverted Bengal gram diet (47). In other exposed to reverted sources, the transferability of microbes has decreased i.e., rice exposed to reverted wheat has transferred 19 genera; soybean exposed to reverted wheat has transferred 12 genera and turmeric exposed to reverted wheat has transferred 5 genera suggesting a decline in genera transferred to reverted phase from their corresponding exposed phase (Fig 7).

**Fig 8:**
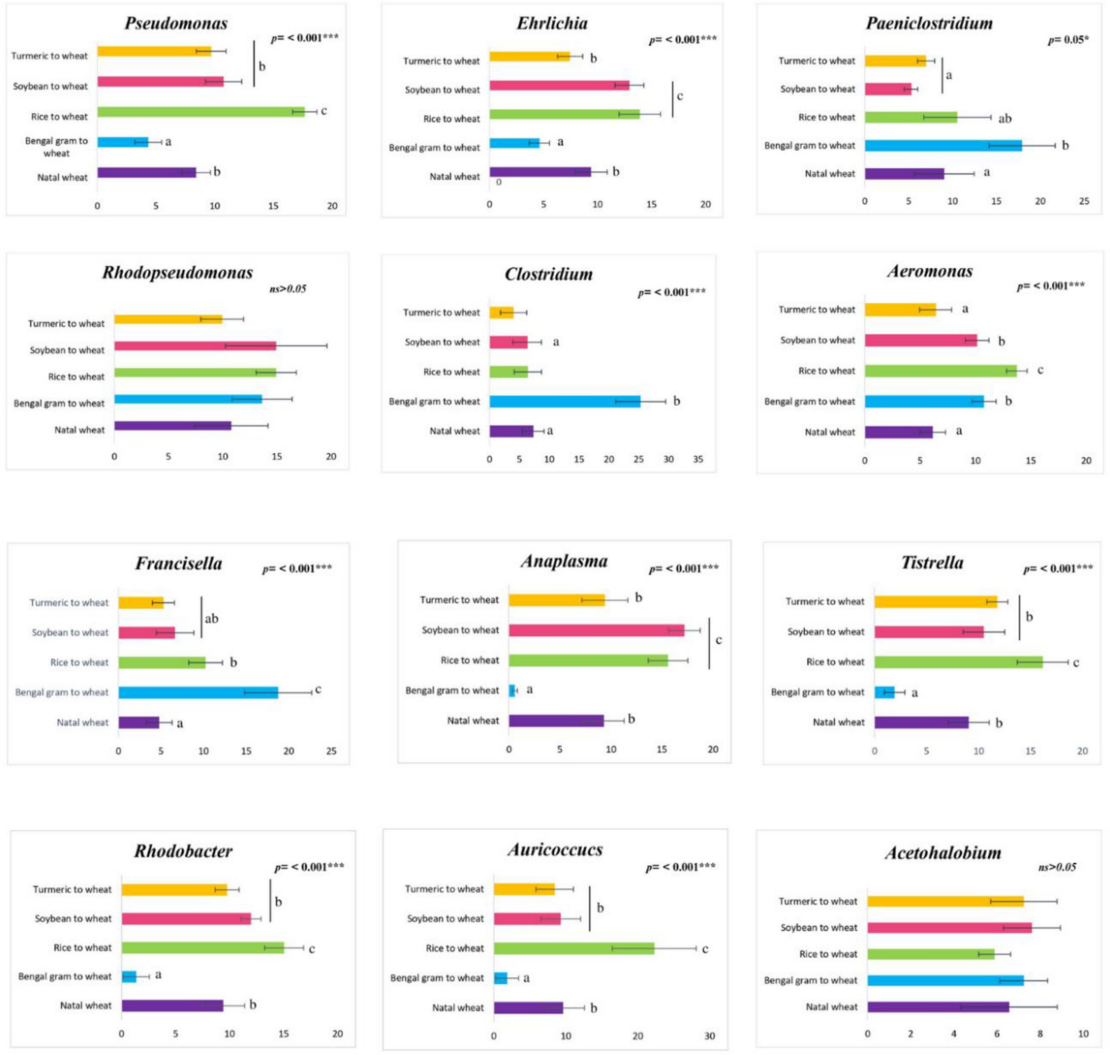
Comparison on the relative abundance of key genera associated with the microbiomes of cigarette beetle from natal phase and reverted phase after six generations. Statistically significant values * = P ≤ 0.05, ** = P ≤ 0.01, *** = P ≤ 0.005; df = 4.

Further, to examine change/shift in abundance of microbiome composition among different phases, multivariant GLM was performed at taxa genus (Table 1). A total of 12 genus exhibited difference among phases belonging to the phylum Proteobacteria and Firmicutes. Each of these taxa (*Pseudomonas*, *Ehrlichia*, *Clostridium*, *Paenioclostridium*, *Aeromonas*, *Rhodopseudomonas*, *Francisella*, *Auricococcus*, *Rhodobacter*, *Anaplasma*, *Tistrella*, and *Acetohalobium)* were exhibiting shift in abundance among phases thereby specifying the plasticity in cigarette beetle microbiome composition (Table 1). Similarly, multivariant GLM between natal and exposed phases revealed the genera *Auricoccus*, *Edwardsiella*, *Paracoccus*, *Acetohalobium*, *Tistrella*, *Rhodopseudomonas*, *Aeromonas*, *Paenioclostridium*, *Anaplasma*, *Ehrlichia*, *Lactobacillus*, *Escherichia* and *Clostridium* to be distinct between the two phases (S7 Table). SIMPER analysis indicated, *Acetohalobium* has contributed an average dissimilarity between natal and exposed phases (S4 Table). In addition, the relative abundance of the selected 12 genera were subjected for univariate GLM analysis to observe for re-assemblage in the microbiome composition between natal wheat and reverted diet sources alone. The abundance of *Acetohalobium* (*F* = 1.464, *p* = 0.284) and *Rhodopseudomonas* (*F* = 1.580; *p* = 0.254) were highly non-significant implying the microbiomes have re-established after being shifted to their natal source (Fig 8). The abundance of genera *Clostridium* were significantly different (*p*≤ 0.01) among natal and reverted sources mainly because of the microbiome prevalence in Bengal gram associated reverted diet (Fig 8).

**Table 1:** Proportion taxa of cigarette beetle (excluding *Wolbachia*) observed among diet sources at different phases and that has contributed > 0.12% to the entire data set at the genus level.

Genus	Proportion natal phase	Proportion exposed phase	Proportion reverted phase	Proportion F-phase†
*Lactococcus*	12.68356	14.60697	12.13535	0.033 ns
*Acetomicrobium*	0.082508	5.412804	3.81402	0.892 ns
*Olleya*	3.354318	7.024463	5.839349	0.808 ns
*Borrelia*	0.267766	4.891328	3.164909	1.116 ns
*Flavobacterium*	2.752433	5.484753	4.305179	0.867 ns
*Sneathia*	1.635573	3.24243	2.700618	0.684 ns
*Chroococcidiopsis*	0.945779	2.424905	1.941365	0.772 ns
*Bacillus*	3.277455	4.632467	2.487438	3.454 ns
*Heliobacterium*	1.127927	1.925634	1.810062	0.274 ns
*Corynebacterium*	1.434324	3.399458	4.66978	0.301 ns
*Mycoplasma*	0.331146	1.368002	1.467619	0.642 ns
*Dialister*	1.971285	1.499923	1.488198	0.349 ns
*Megasphaera*	3.936082	2.438996	2.800256	0.202 ns
*Geminocystis*	0.014499	1.040701	0.675108	1.179 ns
*Bartonella*	3.15228	2.775022	2.673418	0.047 ns
*Enterococcus*	2.957877	1.51247	0.540464	2.315 ns
*Cyanothece*	0.104405	0.808956	0.529245	1.078 ns
*Pseudomonas*	5.996945	1.134732	3.915434	8.783 ***
*Staphylococcus*	0.459766	1.354656	0.849406	0.601 ns
*Gloeocapsa*	0.181659	0.639105	0.47171	1.491 ns
*Truepera*	0.47692	0.700298	0.436851	1.504 ns
*Oscillatoria*	0.593801	0.624832	0.669943	0.049 ns
*Pleurocapsa*	2.195275	0.859854	1.137061	1.116 ns
*Rhodobacter*	4.48284	1.03439	2.574868	6.216 **
*Thermodesulfobium*	0.898531	1.044653	0.911897	0.083 ns
*Burkholderia*	0.728963	2.166598	0.481865	1.552 ns
*Limnochorda*	0.950018	1.038047	0.822586	0.151 ns
*Lactobacillus*	0.792343	0.534057	0.789337	2.322 ns
*Cutibacterium*	0.257089	0.838159	0.351953	1.174 ns
*Prochlorococcus*	0.324604	0.262165	0.339438	0.249 ns
*Ehrlichia*	2.752493	0.500693	1.52519	10.357 ***
*Anaerostipes*	0.499288	0.325161	0.441243	1.332 ns
*Streptococcus*	0.36651	0.573899	0.34317	4.691 ns
*Sodalis*	0.209698	0.260669	0.414867	1.818 ns
*Dichelobacter*	1.482544	0.429204	0.852487	2.434 ns
*Finegoldia*	0.429233	0.319033	0.327934	0.314 ns
*Laceyella*	0.210658	0.358649	0.232889	1.744 ns
*Clostridium*	1.739496	0.261384	0.681838	29.770 ***
*Escherichia*	0.758355	0.330128	0.5311	3.492
*Candidatus Doolittlea*	0	0.241425	0.136621	1.481 ns
*Nitrosomonas*	0.372515	0.254352	0.258422	0.388
*Achromobacter*	0.450017	0.481629	0.337796	0.626 ns
*Anaplasma*	1.981558	0.421055	1.277677	5.583 **
*Candidatus Blochmannia*	0.012108	0.20853	0.133402	1.401 ns
*Ammonifex*	0.041698	1.347826	0.076948	2.383 ns
*Treponema*	0.141393	0.264518	0.247541	1.220 ns
*Vibrio*	0.340934	0.260727	0.375723	1.930 ns
*Planococcus*	0.201341	0.461457	0.220572	1.398 ns
*Methylobacterium*	0.166272	0.291386	0.248401	0.721 ns
*Streptomyces*	0.150446	0.305661	0.360976	0.286 ns
*Thermincola*	0.036396	0.157798	0.116398	0.782 ns
*Chlorobium*	0.137274	0.149177	0.2477	2.754 ns
*Candidatus Xiphinematobacter*	0.012108	0.095663	0.07628	0.419 ns
*Calothrix*	0.065986	0.159199	0.10664	1.414 ns
*Rhodopirellula*	0.454184	0.25252	0.318913	0.339 ns
*Paeniclostridium*	1.038548	0.193398	0.47807	14.903 ***
*Liberibacter*	0.172414	0.181513	1.277055	4.008 ns
*Weissella*	0.170983	0.126426	0.145562	0.319 ns
*Candidatus Pelagibacter*	0.15111	0.167068	0.185699	0.330 ns
*Thermobaculum*	0	0.144883	0.071336	2.646 ns
*Paenibacillus*	0.17503	0.159796	0.157422	0.076 ns
*Herbivorax*	0.102974	0.104276	0.129828	0.260 ns
*Shewanella*	0.200573	0.30245	0.214012	0.508 ns
*Candidatus Sulcia*	0.066282	0.156831	0.2418	1.943 ns
*Aeromonas*	0.698853	0.168383	0.461223	7.776 ***
*Microcystis*	0.056493	0.101384	0.077727	0.400 ns
*Tistrella*	0.900171	0.240301	0.587283	5.320 *
*Rhodopseudomonas*	0.901618	0.117686	0.469097	13.764 ***
*Chryseobacterium*	0.094984	0.141168	0.074474	1.880 ns
*Candidatus Portiera*	0.007398	0.07534	0.06963	0.588 ns
*Desulfotomaculum*	0.163289	0.133269	0.178704	2.213 ns
*Francisella*	0.492587	0.162169	0.305488	8.206 ***
*Pragia*	0.014795	0.102538	0.117911	2.137 ns
*Oxalobacter*	0.124278	0.114808	0.06223	1.625 ns
*Listeria*	0.262177	0.150613	0.16133	1.896 ns
*Paracoccus*	0.473888	0.177896	0.481686	2.751 ns
*Kurthia*	0.012108	0.672441	0.040032	2.209 ns
*Acetohalobium*	0.541058	0.159447	0.292264	3.364 *
*Devosia*	0.077726	0.123073	0.125116	0.655 ns
*Intestinimonas*	0.112691	0.102938	0.085361	0.299 ns
*Edwardsiella*	0.464467	0.110692	0.428663	5.204 ns
*Desulfobacula*	0.082804	0.122429	0.829827	3.345 ns
*Auricoccucs*	0.874793	0.135939	0.402782	9.776 ***
*Turneriella*	0.022193	0.095597	0.050148	2.138 ns
*Pelobacter*	0.041402	0.107187	0.078176	2.441 ns
*Mucilaginibacter*	0.056197	0.074294	0.065878	0.142 ns
*Solibacillus*	0.215072	0.135466	0.115215	1.682 ns

*Note*: Proportional abundance represented by each taxon at different phases are indicated with corresponding *F*‐values (phase) based on multivariate GLM.

†ns = *p* ≥ .05

*p ≤ .05

**p ≤ .01

***p ≤ .001; df (phase) = 2, 24

## Discussion

Cigarette beetle are stored product pests with strong adaptability to diverse food sources from tobacco to edible products, and this may be due to their association with microbial symbionts. Although yeast associated symbiont have been previously reported with cigarette beetle (Martinson 2020), bacterial symbionts association and their dynamics have not been investigated. In the present study, we found support for our hypotheses that bacterial symbionts associated with cigarette beetle exhibit plasticity in accordance with their diet source to help in utilizing the diet. Our results depict the microbiome of cigarette beetle to be significantly influenced by diets and there is re-assemblage in beetle microbiome according to diets. In spite, 12 genera of taxa being distinct among the phases, the microbial abundance of reverted phase was similar to natal phase that reflected the re-assemblage of microbiome in accordance with diet.

### Cigarette beetle microbiome and diversity

Our results indicate the bacterial microbiome of cigarette beetle were dominated by Proteobacteria and Firmicutes at the phylum level (Fig 2), which is consistent with previous studies in insects in general [26, 27] and on beetles [28, 29]. However, the abundance of phylotypes varied consistently among phases and diets especially in exposed and reverted phases of Bengal gram. The bacterial microbiome in Bengal gram diets had a diverse group of communities in both exposed and reverted phase (Fig 2) (i.e. Bacteroidetes, Proteobacteria, Synergistes, Firmicutes, Cyanobacteria and Spirochaetes phylum have equally contributed to the overall Bengal gram associated microbiome diversity). The possibilities for seed microbiome contamination can be ruled out as all the diet sources were irradiated for 15mins before treatment. And the same microbial communities were also observed in other diets and phases but with significantly lower abundance than Bengal gram (Figs 2 and 4). Further, the abundance of Proteobacteria phylum has slightly decreased in exposed phase diets with increase in Firmicutes phylum compared to reverted and natal phase (Fig 2). Similarly, a high microbial diversity and evenness was observed in exposed phase especially with Bengal gram and rice diets (Fig 3). This suggests that the microbial diversity, establishment and proliferation is associated with the diet source of cigarette beetle. This diversification and establishment allows the beetle to utilise various diet with different nutritional composition. A change of diet may induce new species, increasing the diversity and richness of taxa and this can create diet mediated new microbial homeostasis that can be beneficial for the host in assimilating new diet [30]. The diet sources included in the study were diverse with different nutritional values; wheat flour rich in carbohydrates, dietary fibre and protein [31]; whole seed desi Bengal gram seed (brown seed coat) rich in protein (gluteins and prolamines) and high fibre content with low glycemic index along with polysterols and saponins content [32, 33]; soybean flour higher globulin proteins with high digestibility compared to other groups of protein [18]; rice flour rich in carbohydrates with moderate fibre and low protein content [34] turmeric flour rich in secondary metabolites like curcumin [35]. The diverse nutritional composition of diets would have facilitated the establishment of microbiome in response to diet nutrition and needs investigation. The bacterial phyla diversity associated with Bengal gram diet may be involved in digestion of the macromolecules (complex proteins) and provision of nutrients in the beetle. This raises the possibilities that the microbial diversity associated with diets is involved in dietary expansion of the beetle and this requires further detailed investigation.

### *Wolbachia* abundance in cigarette beetle microbiome

*Wolbachia* (Alphaproteobacterium- Anaplasmataceae), is intracellular and maternally inherited and has contributed 84% of the total cigarette beetle microbiome. *Wolbachia* infection is widespread among insects and nearly 50 to 70% insect species are infected with *Wolbachia* [36]. In exposed phases especially with Bengal gram diet, *Wolbachia* abundance is significantly decreased with increase in diversity and establishment of other microbial communities. In natal and reverted phase (excluding Bengal gram), *Wolbachia* abundance was relatively higher in wheat related diets compared to exposed phase diets (Fig 5). The high abundance of *Wolbachia* is mainly related to wheat diet and has suppressed the abundance of other bacterial genera. Wheat diet has been reported as a favourable diet source for cigarette beetle and the beetle can proliferate on wheat at a rapid rate [43; Personal observation]. The proliferation of beetle population on favourable diet suggests the less dependence on microbial symbionts whereas the *Wolbachia* populations acquiring nutrients and carbohydrates from the host for their survival. The host nutrition may have an important role in increase in abundance of *Wolbachia* populations. Previous studies on *Wolbachia* state the endosymbiont to be heavily dependent on the host for nutrition and energy source (Markov and Zakharov 2006). The results from the study provide clues that the abundance of other bacterial genera to be influenced by *Wolbachia* populations. *Wolbachia* infection has an important role in determining the microbial community structure by impeding the diversity and abundance of microbial taxa [37]. In *Drosophila radicum*, *Wolbachia* infection has significantly decreased the diversity and abundance of bacterial genera [36]. In all exposed diet sources the abundance of *Wolbachia* has decreased significantly indicating the microbial cells involved in symbiosis to have re-established for nutrition provision to the host from deficient diet sources. [38] has reported that dietary intake has significantly influenced *Wolbachia* abundance where a high yeast diet has decreased *Wolbachia* abundance and a high sucrose diet has increased *Wolbachia* abundance in female germlines of *Drosophila*.

### Microbiome re-assemblage of cigarette beetle

A change of diet from exposed to the reverted (wheat) phase has shown re-assemblage of microbial composition according to the new diet (Fig 4). The reverted phase exhibited bacterial communities from both exposed and natal phase with variation in abundance (Fig 7). The PCoA (Fig 6) and nMDS plots (S2 Fig) clearly indicate the separation of exposed phase from reverted phase. The reverted phase (expect Bengal gram) are in close occurrence with the natal phase indicating a similarity in bacterial composition between them. However, the exposed phase has an impact on the microbial composition of the reverted phase which can be seen in the venn diagram (Fig 7) where some of the genera associated with the exposed phase is shifted to the reverted phase in the beetle microbiome. The beetle microbiome is dynamic but not transient where the bacterial genera are maintained in the host with the establishment and proliferation of particular genera according to the nutrition requirement and diet source. Prior studies have elaborated that bacterial populations are strongly influenced by host diet and are persistent within the host system and are not transient in nature [39]. Similarly, the results on core genus from venn diagram (Fig 7), SIMPER analysis (S4 and S5 Tables) and heat map on families (Fig 4) indicated a clear stating that the predominant taxa associated with the exposed phase has decreased in abundance in reverted phase and vice versa and are not completely eradicated from the host system (Figs 4 and 7) indicating that when diet conditions favour there is many possibilities that these bacterial cells proliferate to maintain the host health.

In the total and core genera analysed separately, 12 genera exhibited significant changes among the phases belonging to Proteobacteria and Firmicutes (Table 1) phylum denoting the dynamic nature of cigarette beetle microbiome. The changes in microbiota composition in accordance with host diet has been previously reported in Diptera [40–42], Blattodea [43]. The microbiome re-assemblage in reverted phase was evident with *Wolbachia* (Fig 5), *Rhodopseudomonas* and *Acetohalobium* (Fig 8 and S4 Table) genera. These genera have a significantly lower abundance in the exposed phase and have re-established in the reverted phase related to wheat diet. Predictably, the genera have a greater association with wheat based nutrition. The genera *Clostridium* occurred at relatively high abundance in Bengal gram reverted phase and is associated with Bengal gram diet (Fig 8). *Clostridium* genera (Firmicutes), are anaerobic fermenters involved in nutrient provision, production of antimicrobial peptides, and cellulolytic functions [44] and their role in insect symbiosis needs investigation. The study has reported shift in bacterial communities across treatments from cigarette beetle system. However, it should be noted that the study is also limited in using amplicon sequencing for estimating the relative abundance rather than the total bacterial load in the beetle. Information on total microbial density would have helped to make more robust inferences on the nature of observed shifts in microbial community structure and will be investigated in further studies.

## Conclusion

In summary, cigarette beetle microbiome exhibited plasticity in accordance with the diet source. The results indicate that the diet associated host microbiome is a dynamic process and has contributed to the variation observed in insect microbiome. Furthermore, there was re-assemblage of microbial composition in accordance to the diet and the bacterial cells are able to establish and proliferate on reverting to their natal source. This plasticity of microbiome may help the cigarette beetle to utilize diverse foods and expands its food horizon. The results of the study can further lead to novel pest management strategies targeting diet associated microbial plasticity and their ability to utilize food sources. However, in the study the beetle microbiome and its impact on insect fitness have not been systematically analysed. Similarly, other factors that are involved in the diet associated microbiome variance have to be studied and questions on whether the microbiome plasticity is a diet induced response or diet mediated response have to be addressed.

## Supporting information

S1 Table Approximate generation time of cigarette beetle on individual diet sources. (PDF)

S2 Table Summary of proportional abundance of taxa phylum observed on cigarette beetle microbiome from different phases. (PDF)

S3 Table Estimated richness and diversity indices of cigarette beetle for each diet sources across phases. (PDF)

S4 Table SIMPER analysis of cigarette beetle microbial communities showing cumulative percentages of dissimilarity (based on average square-rooted abundance of taxa genus) between significantly different pairs of the factor ‘phases’. (PDF)

S5 Table SIMPER analysis of cigarette beetle microbial communities showing cumulative percentages of dissimilarity (based on average square-rooted abundance of taxa genus) between significantly different pairs of the factor ‘diet sources’. (PDF)

S6 Table Core genus associated with cigarette beetle microbiome across all diet sources and their total abundance. (PDF)

S7 Table Significant values of genus taxa of cigarette beetle (excluding *Wolbachia*) observed among diet sources between natal and exposed phase and that has contributed > 0.12% to the entire data set at the genus level. (PDF)

S1 Fig Rarefaction curve analysis of samples on species richness. (TIF)

S2 Fig Nonmetric multidimensional scaling (MDS) showing ordination of the microbiome communities associated with cigarette beetle across phases. Symbols indicate phases (df = 2).
(TIF)
