# Supplementary material for: Bacterial microbiome associated with cigarette beetle Lasioderma serricorne (F.) and its microbial plasticity in relation to diet sources
Source: PLoS One. 2024 Jan 19;19(1):e0289215. doi: 10.1371/journal.pone.0289215 (PMC10798513; doi:10.1371/journal.pone.0289215)
Supplement: S1 Table — (PDF) [file pone.0289215.s001.pdf]

Diet source	Generation time in days across six generations (approximate)
Wheat	45
Bengal gram	35-40
Rice	50-55
Soybean	45-50
Turmeric	40-45
