# Supplementary material for: Bacterial microbiome associated with cigarette beetle Lasioderma serricorne (F.) and its microbial plasticity in relation to diet sources
Source: PLoS One. 2024 Jan 19;19(1):e0289215. doi: 10.1371/journal.pone.0289215 (PMC10798513; doi:10.1371/journal.pone.0289215)
Supplement: S2 Table — (PDF) [file pone.0289215.s002.pdf]

Phylum	Proportion natal phase	Proportion exposed phase	Proportion reverted phase
Acidobacteria	0.000771	0.006814	0.002289
Actinobacteria	0.128922	1.497595	0.498166
Aquificae	0.002541	0.037223	0.026235
Armatimonadetes	0.000703	0.000301	0.001353
Bacteroidetes	0.299694	6.508091	4.704695
Caldiserica	0.000703	0.002156	0.000419
Calditrichaeota	0	0.004256	0.001353
Candidatus Cloacimonetes	0.001089	0.02448	0.019209
Candidatus Korarchaeota	0.000386	0.00059	0.000451
Candidatus Saccharibacteria	0.002404	0.033136	0.020184
Chlamydiae	0.003926	0.061143	0.039363
Chlorobi	0.009735	0.07711	0.057659
Chloroflexi	0.017462	0.06802	0.02951
Chrysiogenetes	0.001314	0.000802	0.001237
Coprothermobacterota	0.000386	0.002755	0
Crenarchaeota	0.007554	0.046286	0.005616
Cyanobacteria	0.422112	4.684723	2.847637
Deferribacteres	0.001158	0.003469	0.003258
Deinococcus-Thermus	0.018882	0.331723	0.219638
Dictyoglomi	0	0.000301	0.000162
Elusimicrobia	0.000227	0.001378	0.001779
Euryarchaeota	0.008963	0.002675	0.001805
Fibrobacteres	0.001156	0.004422	0.002936
Firmicutes	1.476705	11.12784	4.424902
Fusobacteria	0.068385	1.678246	1.198268
Gemmatimonadetes	0.00433	0.007806	0.002362
Ignavibacteriae	0.001544	0.023931	0.017014
Kiritimatiellaeota	0	0.002373	0.002033
Lentisphaerae	0.000386	0.000868	0.00039
Nitrospirae	0.000544	0.025603	0.017941
Planctomycetes	0.071939	0.219251	0.074892
Proteobacteria	97.39128	65.5961	79.2873
Spirochaetes	0.022281	3.126575	2.510091
Synergistetes	0.003105	3.551125	2.96624
Tenericutes	0.014807	0.985208	0.823305
Thaumarchaeota	0	0.000819	0.000451
Thermodesulfobacteria	0.001771	0.062626	0.042897
Thermotogae	0.008239	0.09594	0.077406
Verrucomicrobia	0.004599	0.096239	0.069553
