# Supplementary material for: Bacterial microbiome associated with cigarette beetle Lasioderma serricorne (F.) and its microbial plasticity in relation to diet sources
Source: PLoS One. 2024 Jan 19;19(1):e0289215. doi: 10.1371/journal.pone.0289215 (PMC10798513; doi:10.1371/journal.pone.0289215)
Supplement: S3 Table — (PDF) [file pone.0289215.s003.pdf]

Phases	Diet	Observed	Chao1	se.chao1	ACE	se.ACE	Shannon	Simpson	Fisher
Natal	Wheat	214	279.2709	20.63132	284.7798	8.570613	1.283724	0.632617	28.65593
Exposed	Bengal gram	319	343.6667	10.67601	342.3452	8.967412	3.480041	0.93848	45.37695
Exposed	Rice	368	383.5833	7.182	382.6173	9.026831	2.68444	0.825671	53.61879
Exposed	Soybean	247	292.8182	15.02439	299.0636	9.570742	1.466628	0.667791	33.71114
Exposed	Turmeric	272	332.8571	18.85698	327.0619	8.303022	1.771803	0.744271	37.69795
Reverted	Bengal gram to wheat	351	369.8571	8.692438	365.9081	8.501105	3.515339	0.941892	50.73334
Reverted	Rice to wheat	212	272.3261	18.28737	290.5598	9.375124	1.337031	0.662921	28.25368
Reverted	Soybean to wheat	208	273	21.37638	257.5542	8.781063	1.278881	0.640189	27.63971
Reverted	Turmeric to wheat	227	271.375	13.83585	304.6437	7.684616	1.31331	0.651793	30.5742
