# Supplementary material for: Bacterial microbiome associated with cigarette beetle Lasioderma serricorne (F.) and its microbial plasticity in relation to diet sources
Source: PLoS One. 2024 Jan 19;19(1):e0289215. doi: 10.1371/journal.pone.0289215 (PMC10798513; doi:10.1371/journal.pone.0289215)
Supplement: S4 Table — (PDF) [file pone.0289215.s004.pdf]

Groups Natal & Exposed						
Average dissimilarity = 9.47						
Genus	Group Natal Av. Abund	Group Exposed Av. Abund	Av.Diss	Diss/SD	Contrib%	Cum. %
Acetohalobium	2.76	2.55	1.1	1.53	11.61	24.46
Anaplasma	4	3.32	0.92	1.06	9.74	34.2
Rhodobacter	4.82	4.19	0.88	1.19	9.3	43.5
Tistrella	3.34	2.89	0.86	1.75	9.09	52.59
Wolbachia	11.57	10.75	0.83	0.91	8.81	61.39
Francisella	2.7	3.56	0.75	1.51	7.89	69.28
Auricoccucs	3.11	2.78	0.64	1.08	6.76	76.05
**Groups Natal & Reverted**						
Average dissimilarity = 8.60						
Genus	Group Natal Av. Abund	Group Reverted Av. Abund	Av.Diss	Diss/SD	Contrib%	Cum. %
Anaplasma	4	3.45	1.02	0.87	11.91	11.91
Auricoccucs	3.11	2.78	0.78	1.12	9.08	31.8
Wolbachia	11.57	11.18	0.78	0.9	9.05	40.85
Rhodobacter	4.82	4.51	0.71	0.92	8.3	49.15
Rhodopseudomonas	3.25	3.47	0.6	2.01	6.98	56.13
Tistrella	3.34	3.18	0.57	1.1	6.63	62.77
Clostridium	3.93	4.01	0.57	1.37	6.63	69.39
Paeniclostridium	3.42	3.57	0.49	1.4	5.67	75.06
**Groups Exposed & Reverted**						
Average dissimilarity = 10.49						
Genus	Group Exposed Av. Abund	Group Reverted Av. Abund	Av.Diss	Diss/SD	Contrib%	Cum. %
Anaplasma	3.32	3.45	1.35	1.14	12.88	12.88
Acetohalobium	2.55	2.82	1.13	2.41	10.81	23.69

Wolbachia	10.75	11.18	1.12	1.18	10.67	34.36
Rhodobacter	4.19	4.51	1.07	1.2	10.23	44.59
Tistrella	2.89	3.18	0.91	1.26	8.66	61.99
Auricoccucs	2.78	2.78	0.88	1.14	8.41	70.4
