# Supplementary material for: Bacterial microbiome associated with cigarette beetle Lasioderma serricorne (F.) and its microbial plasticity in relation to diet sources
Source: PLoS One. 2024 Jan 19;19(1):e0289215. doi: 10.1371/journal.pone.0289215 (PMC10798513; doi:10.1371/journal.pone.0289215)
Supplement: S5 Table — (PDF) [file pone.0289215.s005.pdf]

Diet sources	Average dissimilarity	Genus that maximum contributes to dissimilarity between groups
Wheat and Bengal gram	17.61	*Anaplasma*
Wheat and Bengal gram to wheat	17.97	*Anaplasma*
Bengal gram and Bengal gram to wheat	5.99	*Acetohalobium*
Wheat and rice	9.52	*Acetohalobium*
Bengal gram and rice	13.0	*Acetohalobium*
Bengal gram to wheat and rice	13.41	*Anaplasma*
Wheat and rice to wheat	7.21	*Auricoccus*
Bengal gram and rice to wheat	18.5	*Wolbachia*
Bengal gram to wheat and rice to wheat	19.26	*Anaplasma*
Rice and rice to wheat	11.64	*Acetohalobium*
Wheat and soybean	5.55	*Eschericia*
Bengal gram and soybean	17.43	*Anaplasma*
Bengal gram to wheat and soybean	19.46	*Anaplasma*
Rice and soybean	10.59	*Acetohalobium*
Rice to wheat and soybean	4.2	*Auricoccus*
Wheat and soybean to wheat	4.86	*Eschericia*
Bengal gram and soybean to wheat	18.05	*Anaplasma*
Bengal gram to wheat and soybean to wheat	18.22	*Acetohalobium*
Rice and soybean to wheat	9.84	*Anaplasma*
Rice to wheat and soybean to wheat	4.23	*Auricoccus*
Soybean and soybean to wheat	3.09	*Acetohalobium*
Wheat and turmeric	5.20	*Acetohalobium*
Bengal gram and turmeric	16.71	*Anaplasma*
Bengal gram to wheat and turmeric	18.78	*Anaplasma*
Rice and turmeric	10.01	*Acetohalobium*
Rice to wheat and turmeric	6.14	*Rhodopseudomonas*
Soybean and turmeric	3.39	*Clostridium*
Soybean to wheat and turmeric	4.12	*Acetohalobium*
Wheat and turmeric to wheat	4.37	*Eschericia*

Bengal and turmeric to wheat	17.33	*Wolbachia*
Bengal to wheat and turmeric to wheat	17.69	*Anaplasma*
Rice and turmeric to wheat	9.03	*Acetohalobium*
Rice to wheat and turmeric to wheat	6.78	*Auricoccus*
Soybean and turmeric to wheat	3.95	*Acetohalobium*
Soybean to wheat and turmeric to wheat	3.38	*Anaplasma*
Turmeric and turmeric to wheat	4.36	*Acetohalobium*
