# Supplementary material for: Bacterial microbiome associated with cigarette beetle Lasioderma serricorne (F.) and its microbial plasticity in relation to diet sources
Source: PLoS One. 2024 Jan 19;19(1):e0289215. doi: 10.1371/journal.pone.0289215 (PMC10798513; doi:10.1371/journal.pone.0289215)
Supplement: S6 Table — (PDF) [file pone.0289215.s006.pdf]

Genus	Total abundance
Acetohalobium	222
Acetomicrobium	18444
Achromobacter	584
Acinetobacter	47
Aerococcus	151
Aeromonas	276
Agarivorans	82
Alicyclobacillus	78
Anabaena	110
Anaerolinea	34
Anaerostipes	823
Anaplasma	478
Anoxybacillus	71
Arthrobacter	145
Auricoccucs	192
Bacillus	5802
Bartonella	2529
Borrelia	15195
Bosea	44
Brachyspira	99
Bradyrhizobium	69
Brevundimonas	67
Burkholderia	991
Caldilinea	45
Candidatus Ishikawaella	66
Candidatus Midichloria	109
Candidatus Paracaedibacter	60
Candidatus Pelagibacter	346
Candidatus Profftella	30
Candidatus Walczuchella	43
Chlorobium	387
Chroococcidiopsis	6898
Clostridioides	48
Clostridium	598
Corynebacterium	5517
Coxiella	58
Crinalium	103
Cutibacterium	903
Cyanobacterium	126
Cyanothece	2323
Desulfotomaculum	284
Desulfovibrio	155
Devosia	254

Dialister	3560
Dichelobacter	590
Edwardsiella	210
Ehrlichia	693
Enterococcus	2414
Escherichia	658
Faecalibacterium	47
Finegoldia	696
Flavobacterium	13271
Francisella	267
Gemmatirosa	34
Geobacillus	184
Haemophilus	71
Halanaerobium	83
Heliobacterium	5727
Laceyella	711
Lactobacillus	891
Lactococcus	21221
Liberibacter	364
Limnochorda	934
Listeria	261
Marinitoga	18
Megasphaera	3083
Methylobacterium	447
Microbacterium	158
Microlunatus	17
Moraxella	66
Mucilaginibacter	220
Mycoplasma	5084
Neisseria	80
Nitrospirillum	86
Nocardiopsis	124
Nostoc	197
Olleya	17650
Oscillatoria	1356
Paenibacillus	330
Paeniclostridium	291
Paracoccus	235
Pelobacter	235
Planococcus	496
Pleurocapsa	1142
Propionimicrobium	27
Pseudomonas	1666
Pyrodictium	178

Rhizobium	87
Rhodobacter	1096
Rhodopirellula	331
Rhodopseudomonas	224
Rhodothermus	58
Rubrobacter	27
Ruminococcus	38
Selenomonas	178
Shewanella	308
Sneathia	8273
Sodalis	735
Solibacillus	203
Sphingobacterium	55
Staphylococcus	1787
Stenotrophomonas	168
Streptococcus	794
Streptomyces	454
Tessaracoccus	36
Thermodesulfobium	1171
Tistrella	248
Treponema	538
Truepera	1495
Veillonella	95
Vibrio	515
Vitreoscilla	128
Weissella	353
Wolbachia	825413
Yersinia	114
