# Supplementary material for: Bacterial microbiome associated with cigarette beetle Lasioderma serricorne (F.) and its microbial plasticity in relation to diet sources
Source: PLoS One. 2024 Jan 19;19(1):e0289215. doi: 10.1371/journal.pone.0289215 (PMC10798513; doi:10.1371/journal.pone.0289215)
Supplement: S7 Table — (PDF) [file pone.0289215.s007.pdf]

S. No.	Genus	F value	Sig. value
1	*Lactococcus*	0.013589	0.908981
2	*Acetomicrobium*	1.966302	0.184266
3	*Olleya*	1.601404	0.227916
4	*Borrelia*	2.325124	0.151253
5	*Flavobacterium*	1.423243	0.25419
6	*Sneathia*	1.256241	0.282642
7	*Chroococcidiopsis*	1.61844	0.225595
8	*Bacillus*	0.624568	0.443533
9	*Heliobacterium*	0.458636	0.510135
10	*Corynebacterium*	0.302401	0.591704
11	*Mycoplasma*	1.038285	0.32681
12	*Dialister*	0.493712	0.494667
13	*Megasphaera*	0.302264	0.591787
14	*Geminocystis*	2.498337	0.137982
15	*Bartonella*	0.050758	0.825252
16	*Enterococcus*	0.808703	0.384859
17	*Cyanothece*	2.08018	0.17288
18	*Pseudomonas*	21.50434	0.000465
19	*Staphylococcus*	0.522841	0.48244
20	*Gloeocapsa*	3.048117	0.104403
21	*Truepera*	0.702865	0.416972
22	*Oscillatoria*	0.009082	0.92553
23	*Pleurocapsa*	1.680158	0.217443
24	*Rhodobacter*	11.24609	0.005186
25	*Thermodesulfobium*	0.058529	0.81261
26	*Burkholderia*	0.466123	0.506761
27	*Limnochorda*	0.016604	0.899444
28	*Lactobacillus*	5.679064	0.033117 *
29	*Cutibacterium*	0.691519	0.420661
30	*Prochlorococcus*	0.096934	0.760477
31	*Ehrlichia*	30.49683	9.83E-05 *
32	*Anaerostipes*	1.880234	0.193514
33	*Streptococcus*	2.307697	0.152675
34	*Sodalis*	0.218015	0.648285
35	*Dichelobacter*	4.527488	0.053063
36	*Finegoldia*	0.498105	0.492788
37	*Laceyella*	1.419997	0.254705
38	*Clostridium*	57.07072	4.16E-06*
39	*Escherichia*	7.646867	0.016061*
40	*Candidatus Doolittlea*	2.867105	0.114218
41	*Nitrosomonas*	0.590883	0.455817
42	*Achromobacter*	0.016751	0.899003
43	*Anaplasma*	12.76623	0.003403*
44	*Candidatus Blochmannia*	2.781397	0.119259
45	*Ammonifex*	0.971189	0.342371
46	*Treponema*	2.079883	0.172908
47	*Vibrio*	0.935338	0.351129

48	*Planococcus*	0.655255	0.432807
49	*Methylobacterium*	1.100931	0.313178
50	*Streptomyces*	0.407435	0.534358
51	*Thermincola*	1.813548	0.201093
52	*Chlorobium*	0.025368	0.875902
53	*Candidatus Xiphinematobacter*	0.80141	0.386948
54	*Calothrix*	1.542198	0.236229
55	*Rhodopirellula*	0.471406	0.504404
56	*Paeniclostridium*	30.09307	0.000105*
57	*Liberibacter*	0.003677	0.952568
58	*Weissella*	0.550387	0.471351
59	*Candidatus Pelagibacter*	0.087321	0.772278
60	*Thermobaculum*	4.82868	0.046718
61	*Paenibacillus*	0.211695	0.653043
62	*Herbivorax*	0.000534	0.981913
63	*Shewanella*	0.334101	0.573131
64	*Candidatus Sulcia*	2.345857	0.149583
65	*Aeromonas*	15.20291	0.001829*
66	*Microcystis*	0.667796	0.428544
67	*Tistrella*	12.16366	0.004008*
68	*Rhodopseudomonas*	29.50668	0.000115*
69	*Chryseobacterium*	0.762412	0.398417
70	*Candidatus Portiera*	1.020079	0.33093
71	*Desulfotomaculum*	1.290861	0.276401
72	*Francisella*	19.52864	0.000693*
73	*Pragia*	3.954231	0.068227
74	*Oxalobacter*	0.035275	0.85392
75	*Listeria*	2.993747	0.107238
76	*Paracoccus*	6.022338	0.028992*
77	*Kurthia*	0.922764	0.354277
78	*Acetohalobium*	6.354363	0.025566*
79	*Devosia*	1.283196	0.277766
80	*Intestinimonas*	0.043676	0.837697
81	*Edwardsiella*	11.61049	0.004675*
82	*Desulfobacula*	0.122192	0.732266
83	*Auricoccucs*	15.78662	0.00159*
84	*Turneriella*	3.369181	0.0894
85	*Pelobacter*	3.507965	0.083733
86	*Mucilaginibacter*	0.2313	0.638555
87	*Solibacillus*	1.692358	0.215877
