# Supplementary figures and images for: Bacterial microbiome associated with cigarette beetle Lasioderma serricorne (F.) and its microbial plasticity in relation to diet sources
Source: PLoS One. 2024 Jan 19;19(1):e0289215. doi: 10.1371/journal.pone.0289215 (PMC10798513; doi:10.1371/journal.pone.0289215)

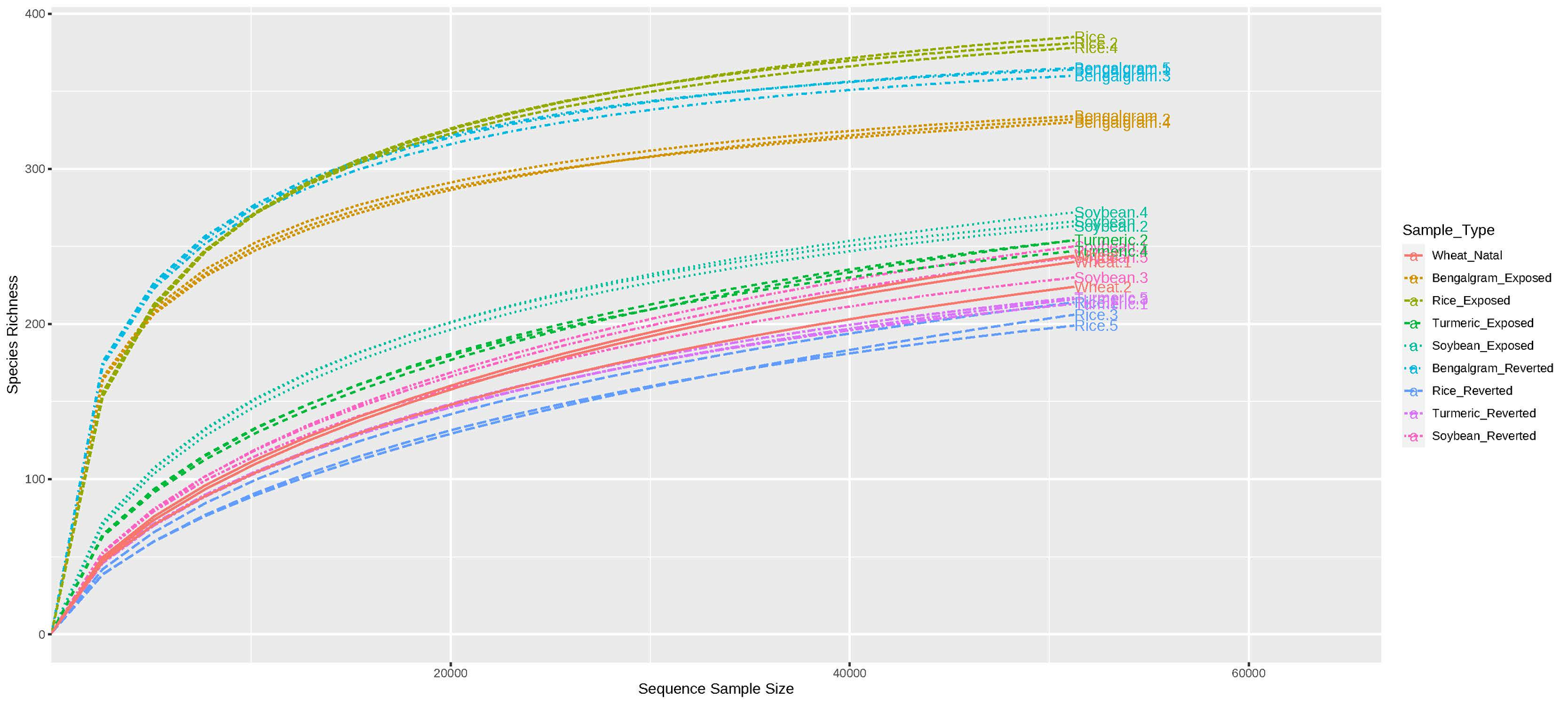

Supplement: S1 Fig — (TIF) [file pone.0289215.s008.tif]

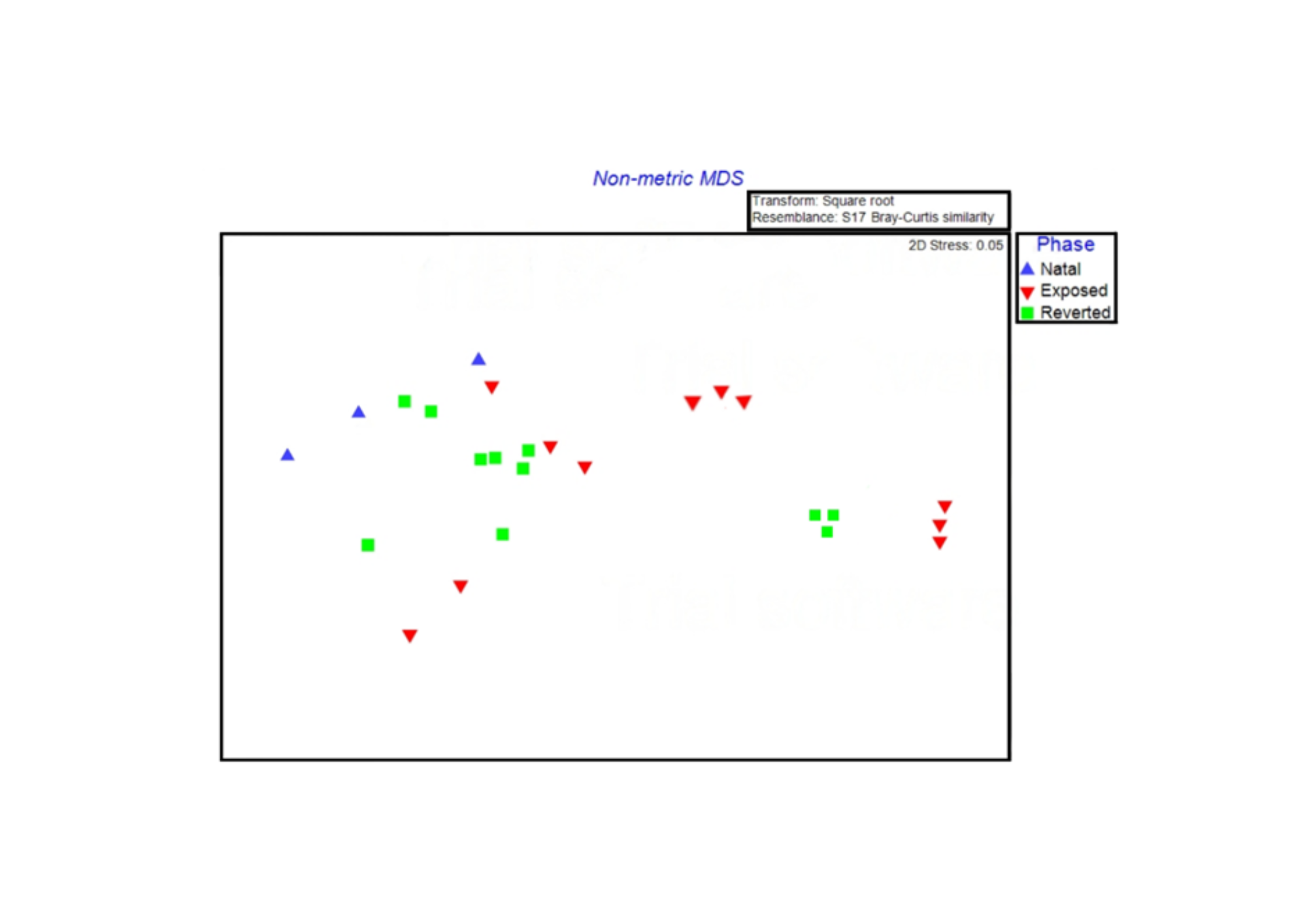

Supplement: S2 Fig — Symbols indicate phases (df = 2). (TIF) [file pone.0289215.s009.tif]
